# Analyzing experimental data from reciprocating wear testing on piston aluminum alloys, with and without clay nano-particle reinforcement

**DOI:** 10.1016/j.dib.2022.108766

**Published:** 2022-11-21

**Authors:** Mohammad Azadi, Ali Shahsavand, Mohammad Sadegh Aghareb Parast

**Affiliations:** Faculty of Mechanical Engineering, Semnan University, Semnan, Iran

**Keywords:** Wear dataset, Wear rate, Friction coefficient, Aluminum alloys, Nano-particles, Nano-composite

## Abstract

In the present experimental data, reciprocating wear testing was done on piston aluminum alloys. In some cases, this material was also reinforced by 1% wt. of clay nano-particles and also tested under wear conditions. For this objective, a permanent-mold casting process was done for the aluminum alloy sample. Besides, a stir-casting technique was used for the fabrication of aluminum-matrix nano-composite plus preheating of nano-particles. Then, for both material types (aluminum alloys, with and without nano-particle reinforcement), the weight, the wear rate, and the friction coefficient were measured during testing. Reciprocating wear testing was performed based on the ASTM-G133 standard for 500 m of the wear distance. Other factors were considered as 10, 20, and 30 N for the applied force with a linear velocity of 1 and 7 m/s (equal to 600 and 3600 rpm of the wear testing device). A nodular cast iron (MF-116) based on the piston ring material was utilized as the abrasive system with a hardness of 35–45 HRC in a dry environment. Finally, obtained experimental results were analyzed by a regression technique for the sensitivity analysis of outputs on inputs. Three input parameters were the force, the velocity, and the reinforcement. Moreover, the total wear rate and the average friction coefficient were the output factors. The effect of each input on all outputs was drawn in different contour and surface diagrams.

**Specifications Table**

**Table:** 

Subject	Engineering
Specific subject area	Engineering/ Mechanical Engineering/ Automotive Engineering
Type of data	Table Image Figure
How the data were acquired	A reciprocating wear testing device was considered to obtain the wear behavior of as-cast and nano-reinforced aluminum samples. During experiments, the specimen weight was measured to find the wear rate. Moreover, the contact force was also measured to determine the friction coefficient. Finally, a sensitivity analysis was carried out to obtain the influence of inputs (the applied force, the linear velocity, and the reinforcement) on the wear behavior of piston aluminum alloys.
Data format	Raw Analyzed
Description of data collection	Standard samples from piston aluminum alloys with and without clay nano-particle reinforcement were tested under reciprocating wear loading conditions as follows: Distance: 500 m Applied forces: 10, 20, and 30 N Linear velocity: 1 and 7 m/s (equal to 600 and 3600 rpm) Abrasive system: A nodular cast iron (MF-116) ring with a hardness of 35–45 HRC in a dry environment (without oil or lubricant).
Data source location	Institution: Semnan University, Faculty of Mechanical Engineering, Research Laboratory of Advanced Materials Behavior (AMB) City/Town/Region: Semnan Country: Iran Latitude and longitude (and GPS coordinates, if possible) for collected samples/data: 35.59878671018807, 53.433229370400255
Data accessibility	Repository name: Mendeley Data Data identification number (permanent identifier, i.e., DOI number): 10.17632/my3fx9wzyb.2 Direct link to the dataset: https://data.mendeley.com/datasets/my3fx9wzyb/2

## Value of the Data

• One important issue in vehicle engines is the piston-ring component under wear conditions in the cylinder block. This material behavior affects the engine performance and also environmental emissions and fuel consumption. Consequently, knowing the tribological behavior of aluminum alloys in the engine piston is so essential for design engineers in the automotive industry.
• One effective factor in the tribological behavior of the material is the wear rate. Knowing how much the wear rate of the engine component is under working conditions is also necessary for designers. The other significant parameter is the friction coefficient between the piston and the ring with different material types. This issue will directly affect the fuel consumption of the vehicle, since the friction power will be lost in the engine, from the initial power of the fuel. If the designer could reduce this frictional work, they have a superior output.
• One novel technique to have a better wear behavior of the material is nano-technology, which could lead to an improvement in the wear rate and the friction coefficient. This enhanced strength could be occurred for aluminum alloys by adding nano-particles through the casting process (stir-casting). Besides, knowing the influences of the applied force and the linear velocity during the wear phenomenon could help the engine engineers to have an optimum design for the piston-ring component.
• These data provide crucial information about materials used in parts exposed to wear and alternative loads and speeds. In addition, the mentioned data can use to improve the lifetime of the contacting industrial components. Moreover, these data can be helpful for all engine components manufacturers who seek lightweight materials with proper wear behavior, especially piston manufacturing.
• The obtained results can lead to the extraction of the material properties, which is crucial in further CAE applications.

## 1 Data Descriptions

As the first description for the experimental data, an excel file including the raw data is available at https://data.mendeley.com/datasets/my3fx9wzyb/2 in the Mendeley data [1]. This file includes the wear testing results based on inputs (the speed, the force, and the nano reinforcement) and outputs (the average coefficient of friction (CoF) and the total wear rate in one sheet, entitled “All Data” and also, the CoF during 500 m and the wear rate at every 100 m in two other sheets, entitled “CoF” and “Wear Rate”).

**Fig. 1:**
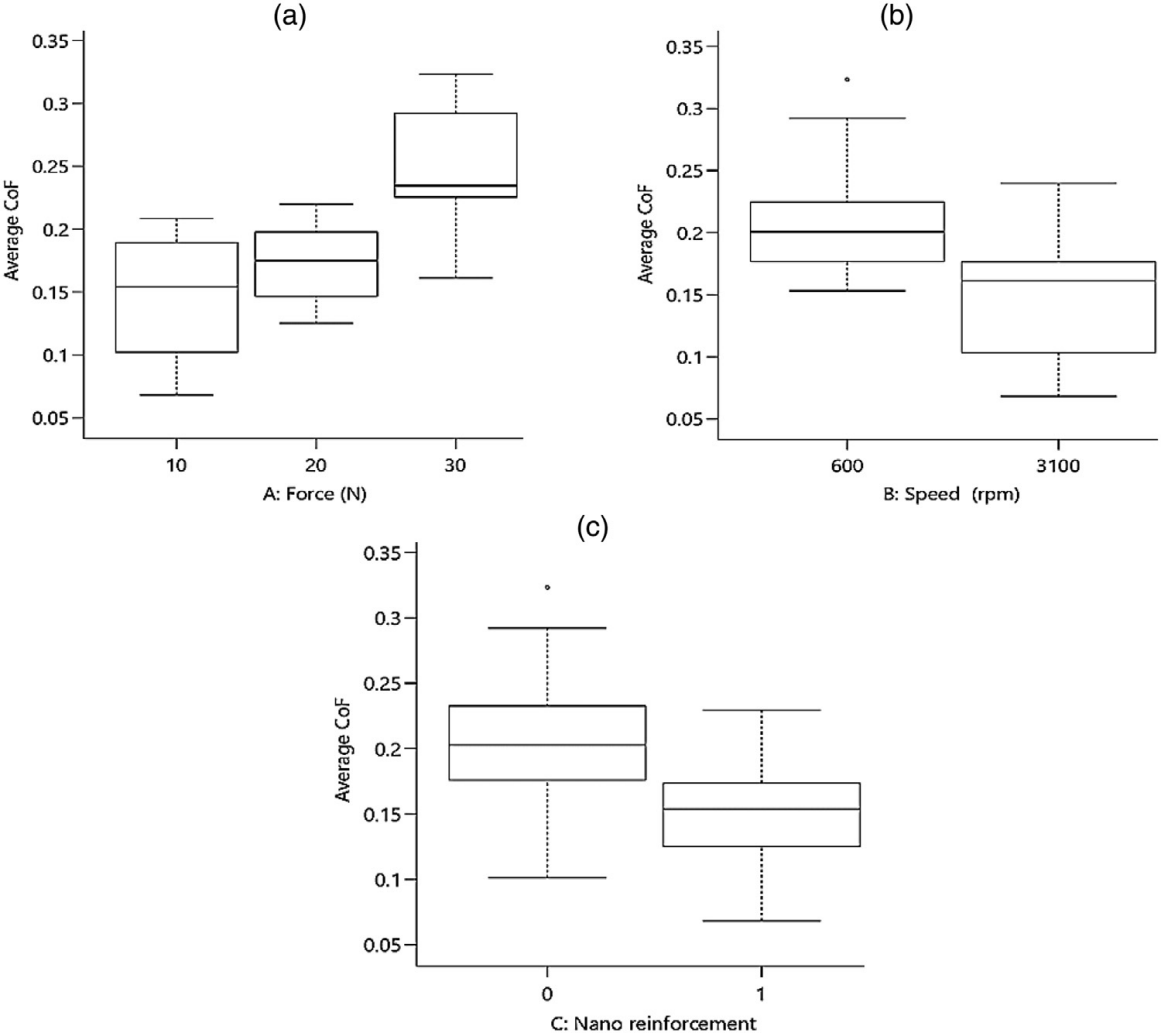
The scatter-band of the average CoF for different inputs (In the third part, “0” is for the piston aluminum alloy and “1” is for the nano-composite.).

**Fig. 2:**
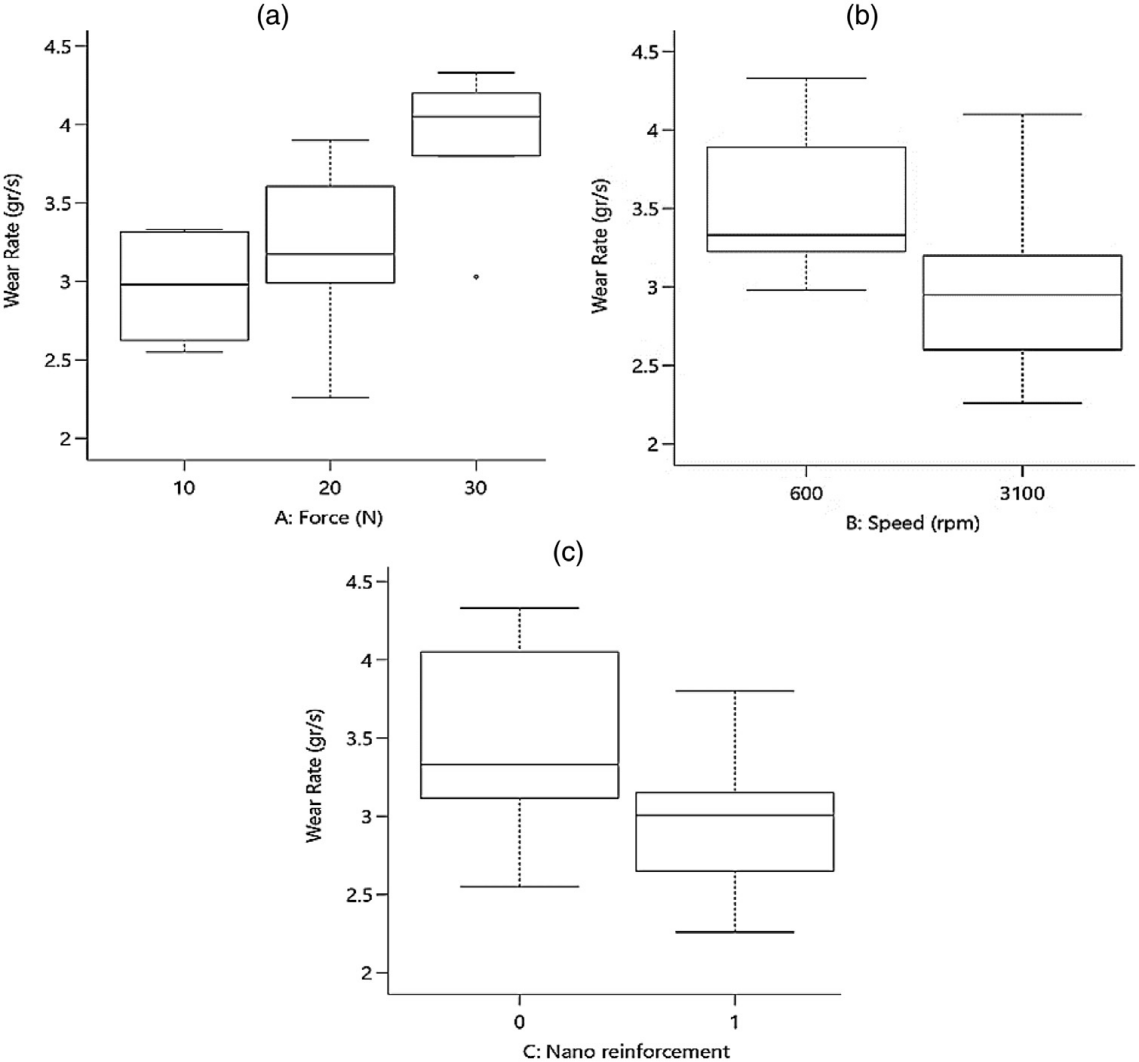
The scatter-band of the total wear rate (× 10^−4^) for different inputs (In the third part, “0” is for the piston aluminum alloy and “1” is for the nano-composite.).

To check the scatter-band (or the variation) of the outputs versus the inputs, Figs. 1 and 2 present the average CoF and the total wear rate, respectively for piston aluminum alloys.

As it could be seen from the results for the average CoF, by increasing the applied force, the COF value increased, as expected. However, this behavior was reversed when the speed was enhanced. With the addition of nano-particles, the average CoF of the piston aluminum alloy decreased, which illustrated an improvement in the wear behavior of the material. A similar result could be observed for the total wear rate in the material, compared to the effect of CoF. In other words, nano-particles improved the total wear rate besides the lower value of CoF. The applied force led to an increase in the total wear rate and the speed caused to decrease in this output.

Nuruzzaman and Chowdhury [2] illustrated that for different aluminum alloys, increasing the normal load caused to increase in the CoF during pin-on-disk wear testing, which agrees with the results of the present data. However, the CoF increased when the sliding velocity enhanced due to more adhesion of the counterface material (pin) on the aluminum alloys [2]. Chen et al. [3] and Singhal and Pandey [4] indicated that the amount of force will have a direct connection with the wear rate and CoF; therefore, at higher forces, these two factors will be also higher. Ramesh and Ahamed [5] also achieved similar results that the wear rate increased with the increase in load. On the contrary, Leon-Patino et al. [6] reported a mixed behavior on this issue for Al-Ni/SiC composites. They demonstrated that the higher wear velocity led to lower wear intensity in the material. Moreover, during pin-on-disk wear testing at different distances, various tribological behaviors were seen under different velocities and the wear modes changed [6]. The data in the present work was in an agreement with the results of Leon-Patino et al. [6].

Huang et al. [7] showed SiC particles (by centrifugal casting) could improve the wear resistance of the piston hypereutectic aluminum-silicon alloy. In another work, Choi et al. [8] demonstrated that carbon nanotubes were an effective reinforcement to enhance the wear properties as well as mechanical properties of aluminum alloys. Similarly, Karbalaei Akbari et al. [9] observed that the higher amount of nano-particles in the matrix increased the wear-resistant and stability. Finally, Azadi et al. [10,11] suggested using SiO_2_ nano-particles with the T6 heat treatment in order to increase the wear resistance of the piston aluminum alloy. From these works, it could be concluded that such reinforcements could enhance the wear strength of the piston aluminum alloy, which also occurred in this data, as in agreement with the literature [7], [8], [9], [10], [11], but with a different nano-particle (clay).

Notably, these descriptions are qualitative results, and therefore, by performing a linear/nonlinear regression analysis, a quantitative result could be in the next section for better understanding of the material behavior. This action was done by the Design-Expert software.

Design-Expert performs a Shapiro-Wilk hypothesis test on the normality of the unselected terms on the effects plot. The null hypothesis is that the unselected terms represent noise. Then, noise is assumed to follow a normal distribution. When the selection of statistically significant terms is complete, the Shapiro-Wilk P-value should above 0.10 to indicate there is no significant deviation from the assumption of normality for the non-selected effects. Notably, the Shapiro-Wilk test for normality is not shown on the normal probability plot of residuals, since this plot violates the assumption of independence by ordering the residuals.

Before finalizing the regression model, different models are compared in Table 1, including linear, 2FI (Two Factor Interaction), and quadratic ones, besides the evaluation of each model. The selected model must have the maximum values of adjusted R² and the predicted R², in addition to having a negligible lack-of-fit. According to Table 1, the linear model was proper for the average COF and the 2FI model was proper for the wear rate.

**Table 1:** The results for the initial tests of the regression analysis.

Source	Seq. *P*-value	Lack of Fit	Sum of Squares	Mean Square	*P*- Value	Std. Dev.	*R*²	Adjusted *R*²	Predicted *R*²	Suggestion
**Response: Average COF**										
Linear	< 0.0001	0.490	0.646	0.080	0.058	0.021	0.891	0.874	0.840	YES
2FI	0.7164	0.330	0.327	0.065	0.118	0.022	0.900	0.862	0.791	NO
Quadratic	0.0927	0.520	0.243	0.060	0.148	0.021	0.917	0.879	0.794	NO
**Response: Wear Rate**										
Linear	< 0.0001	0.050	0.646	0.080	0.058	0.225	0.867	0.846	0.804	NO
2FI	0.0852	0.120	0.327	0.065	0.119	0.200	0.911	0.877	0.807	YES
Quadratic	0.1525	0.150	0.243	0.060	0.148	0.193	0.922	0.886	0.809	NO

Based on the results of the Design-Expert software, Table 2 demonstrates the sensitivity analysis for the average CoF. Moreover, these results are repeated for the total wear rate of the piston aluminum alloy in Table 3. In addition, Table 4 presents the accuracy of the regression analysis for both outputs. In these tables, the source (including the model and different parameters), summation of squares, the degree of freedom (df), mean square, the *F*-Value, the *P*-Value, and the effectiveness are mentioned. For the accuracy of fitting, the standard deviation (Std. Dev.), mean, C.V. %, and values for the coefficient of determination (*R*^2^) are reported.

**Table 2:** The results of analyzed data for the average COF of the piston aluminum alloy.

Source	Sum of Squares	df	Mean Square	*F*-Value	*P*-Value	Effectiveness
Model	0.0683	3	0.0228	52.00	< 0.0001	Significant
A: Force	0.0302	1	0.0302	69.06	< 0.0001	Significant
B: Speed	0.0209	1	0.0209	47.80	< 0.0001	Significant
C: Nano reinforcement	0.0145	1	0.0145	33.16	< 0.0001	Significant
Residual	0.0083	19	0.0004	-	-	-
Lack of Fit	0.0035	8	0.0004	0.9929	0.4902	Non-significant
Pure Error	0.0048	11	0.0004	-	-	-
Cor Total	0.0766	22	-	-	-	-

**Table 3:** The results of analyzed data for the total wear rate (× 10^−4^) of the piston aluminum alloy.

Source	Sum of Squares	df	Mean Square	*F*-Value	*P*-Value	Effectiveness
Model	6.59	6	1.10	27.27	< 0.0001	Significant
A: Force	2.46	1	2.46	61.08	< 0.0001	Significant
B: Speed	1.66	1	1.66	41.23	< 0.0001	Significant
C: Nano reinforcement	1.48	1	1.48	36.68	< 0.0001	Significant
AB	0.0084	1	0.0084	0.2093	0.6534	Non-significant
AC	0.3134	1	0.3134	7.78	0.0131	Significant
BC	2.140E-06	1	2.140E-06	0.0001	0.9943	Non-significant
Residual	0.6442	16	0.0403	-	-	-
Lack of Fit	0.3275	5	0.0655	2.28	0.1189	Non-significant
Pure Error	0.3167	11	0.0288	-	-	-
Cor Total	7.23	22	-	-	-	-

**Table 4:** The accuracy of the regression analysis for the piston aluminum alloy.

Parameters	Average CoF	Total wear rate
Std. Dev.	0.0209	0.2007 × 10^−4^
Mean	0.1825	3.2900 × 10^−4^
C.V. %	11.46	6.09
*R*²	89.14%	91.09%
Adjusted *R*²	87.43%	87.75%
Predicted *R*²	84.03%	80.69%
Adeq. Precision	23.23	17.36

As the first observation from the nonlinear regression analysis, the model was significant for both outputs, the average CoF and the total wear rate. The *P*-Value was less than 0.05, equal to 95% of the confidence level. Moreover, the *R*^2^ value was 89.14% and 91.09% for the average CoF and the total wear rate, respectively. In addition, the *P*-Value for the lack of fit was higher than 0.05 and consequently, it was not significant for both outputs. These issues mean that the regression analysis was meaningful and the model was proper for the prediction of the material behavior.

For the total wear rate, the predicted *R*^2^ of 80.69% was in reasonable agreement with the adjusted *R*^2^ of 87.75%; i.e. the difference was less than 0.2. For the average CoF, the predicted *R*^2^ of 84.03% was also in proper agreement with the adjusted *R*² of 87.43%; i.e. the difference was less than 0.2. The Adeq. precision measures the signal to noise ratio. A ratio greater than 4 is desirable. This ratio of 23.23 and 17.36 for the average CoF and the total wear rate, respectively, indicated an adequate signal for both outputs.

All inputs had an effective influence on both outputs through the linear regression analysis. However, unless one case (AC for the total wear rate in Table 3), other interactions (AB, AC, and BC) or other nonlinear terms between inputs had no significant effect on outputs.

Based on the *F*-Value in Tables 2 and 3, the applied force was the most effective parameter on both outputs, which had the highest amount of the *F*-Value. The nano-particle reinforcement had the lowest *F*-Value among three inputs in the linear regression.

The mentioned models for the linear regression for two outputs ($COF_{avg}$: average CoF and $WR_{tot}$: total wear rate) versus the inputs (A: force, B: speed, and C: nano reinforcement) are as follows. The low value of coefficients in the regression model leads to the elimination of the related term in the following equations.

(1) $$COF_{avg}\;\left(for\;aluminum\;alloy\right)=0.16264+0.00456A-0.00002B$$

(2) $$COF_{avg}\;\left(for\;nano-composite\right)=0.11178+0.00456A-0.00002B$$

(3) $$WR_{tot}\;\left(for\;aluminum\;alloy\right)=\left(2.88543+0.05392A-0.000259B\right)\times 10^{-4}$$

(4) $$WR_{tot}\;\left(for\;nano-composite\right)=\left(2.96641+0.023666A-0.00025B\right)\times 10^{-4}$$

Notably, in Eqs. (1)–(4), the term “C”, which indicates whether the nano reinforcement existed or not in the material, was not reported directly because of the style of the coded equation, besides the actual equation. Then, this issue is considered by dividing into two formulations of Eq. (1) (for aluminum alloy) and 2 (for nano-composite), similar to Eqs. (3) and (4).

**Fig. 3:**
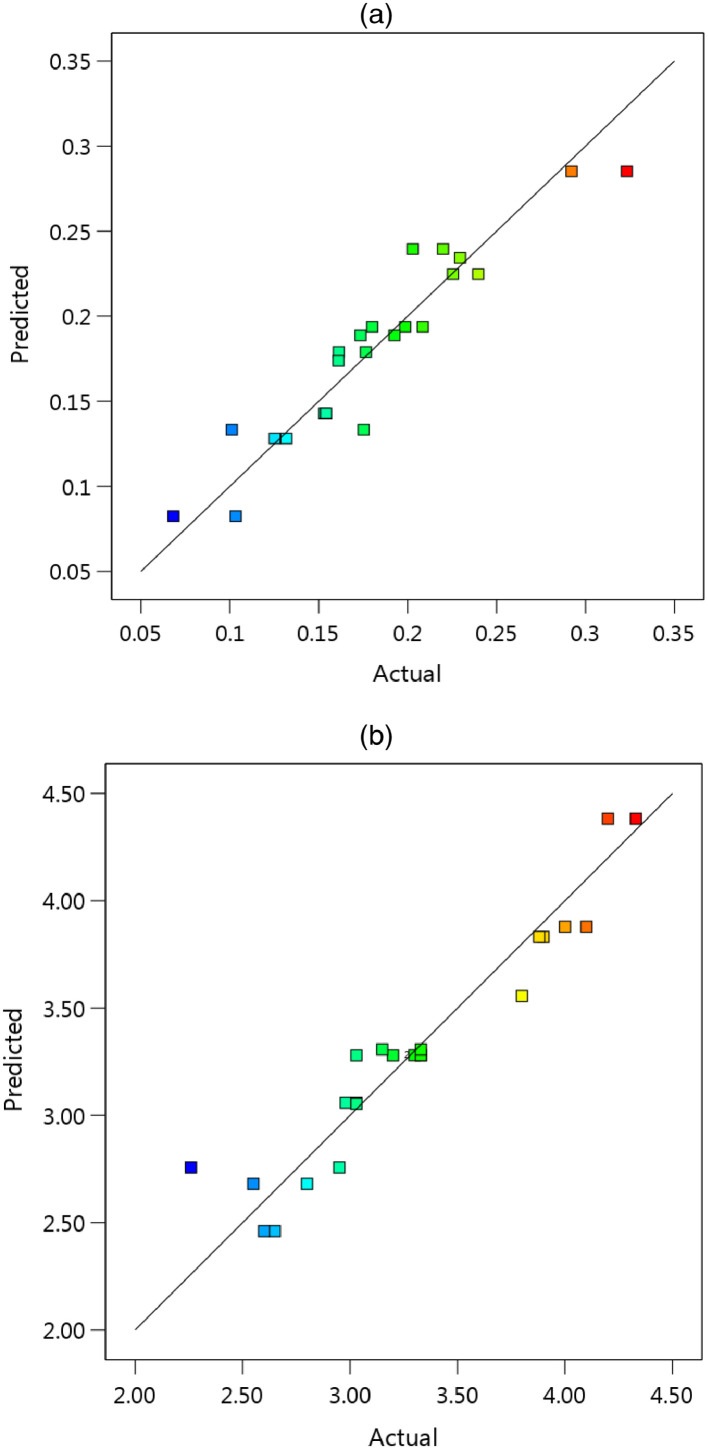
The scatter-band of the experimental (actual) and predicted values for (a) the average COF and (b) the total wear rate (× 10^−4^).

Using the above formulations, the scatter-band for the predicted versus experimental (actual) values could be drawn, as depicts in Figure 3 for both outputs. As a concluded mark, it could be claimed that the scatter-band was similar for the average COF and the total wear rate.

**Fig. 4:**
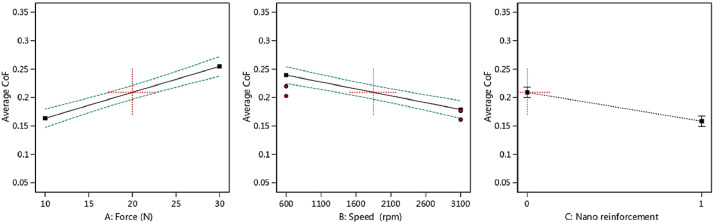
The trend of the average CoF versus input parameters in the piston aluminum alloy.

**Fig. 5:**
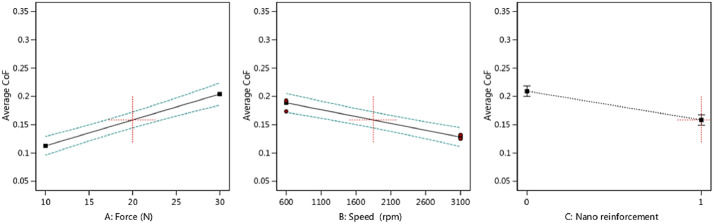
The trend of the average CoF versus input parameters in the nano-composite.

**Fig. 6:**
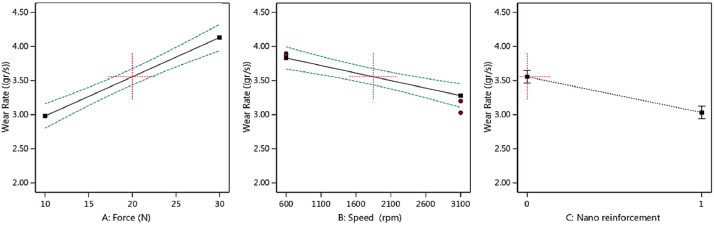
The trend of the total wear rate (× 10^−4^) versus input parameters in the as-cast sample.

**Fig. 7:**
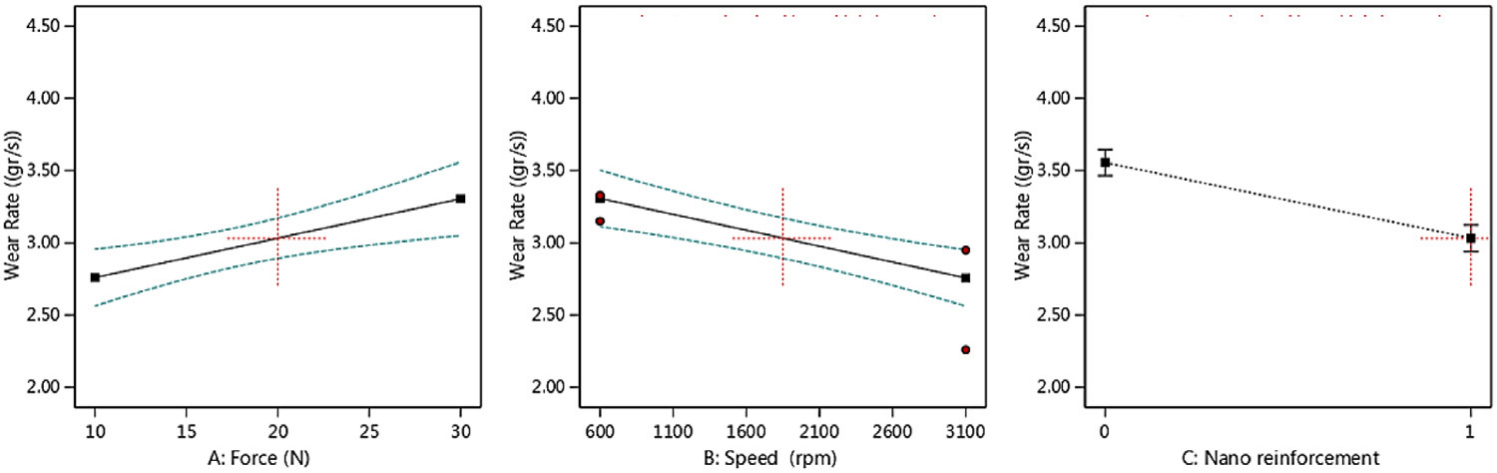
The trend of the total wear rate (× 10^−4^) versus input parameters in the nano-composite.

Then, the output trend could be observed in Fig. 4, Fig. 5, Fig. 6, Fig. 7 versus the input variables for both materials, including the piston aluminum alloy and the nano-composite. In both cases, as mentioned before, by increasing the force, the average CoF and the total wear rate increased. These behaviors were reversed for the speed. Moreover, the tribological behavior of the nano-composite was better than the piston aluminum alloy, since the total wear rate and the average CoF were lower when clay nano-particles were added to the aluminum matrix.

**Fig. 8:**
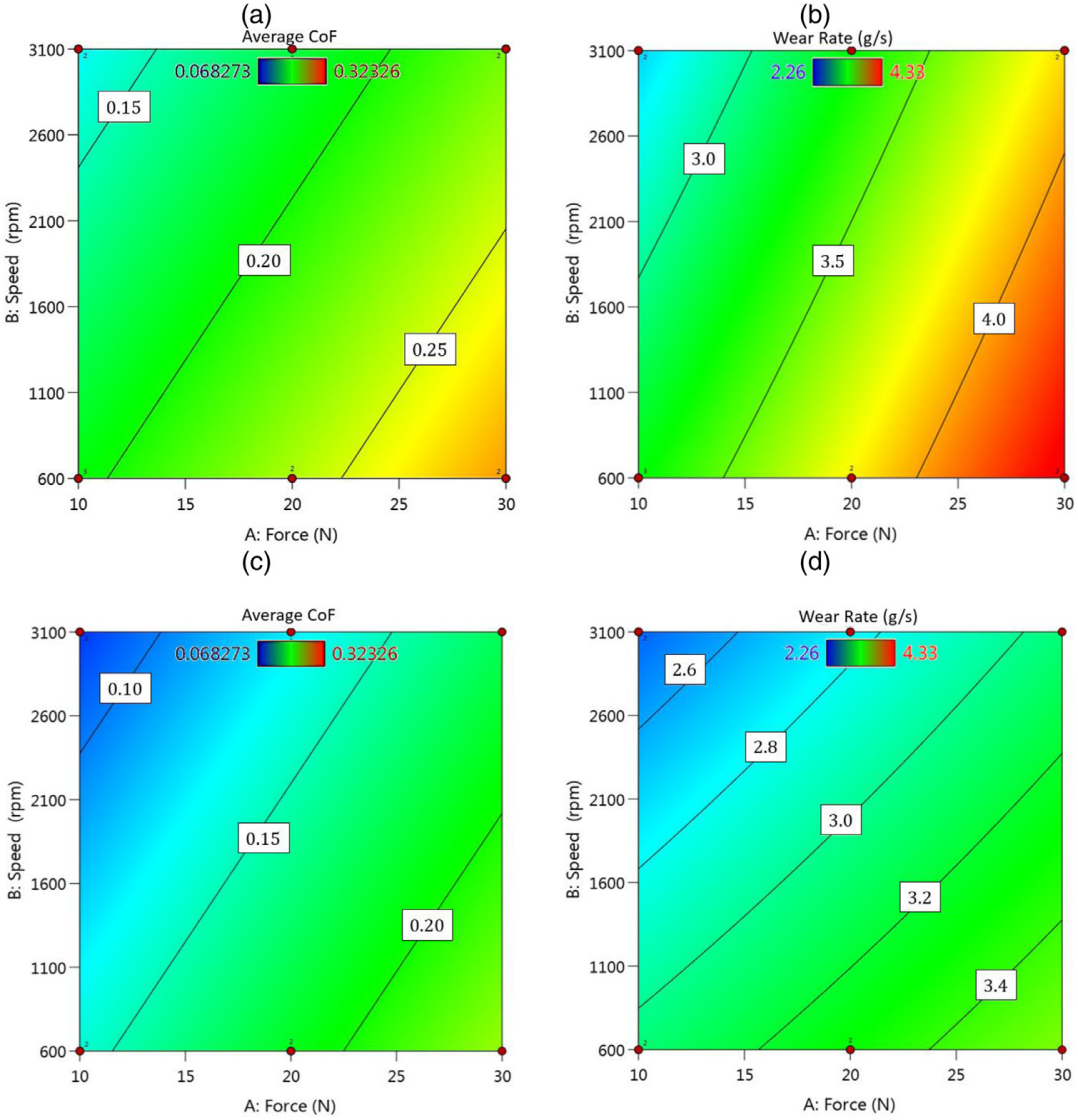
The contour plots of outputs (the average CoF and the total wear rate × 10^−4^) versus the speed and the force: (a) and (b) for the piston aluminum alloy and (c) and (d) for the nano-composite.

**Fig. 9:**
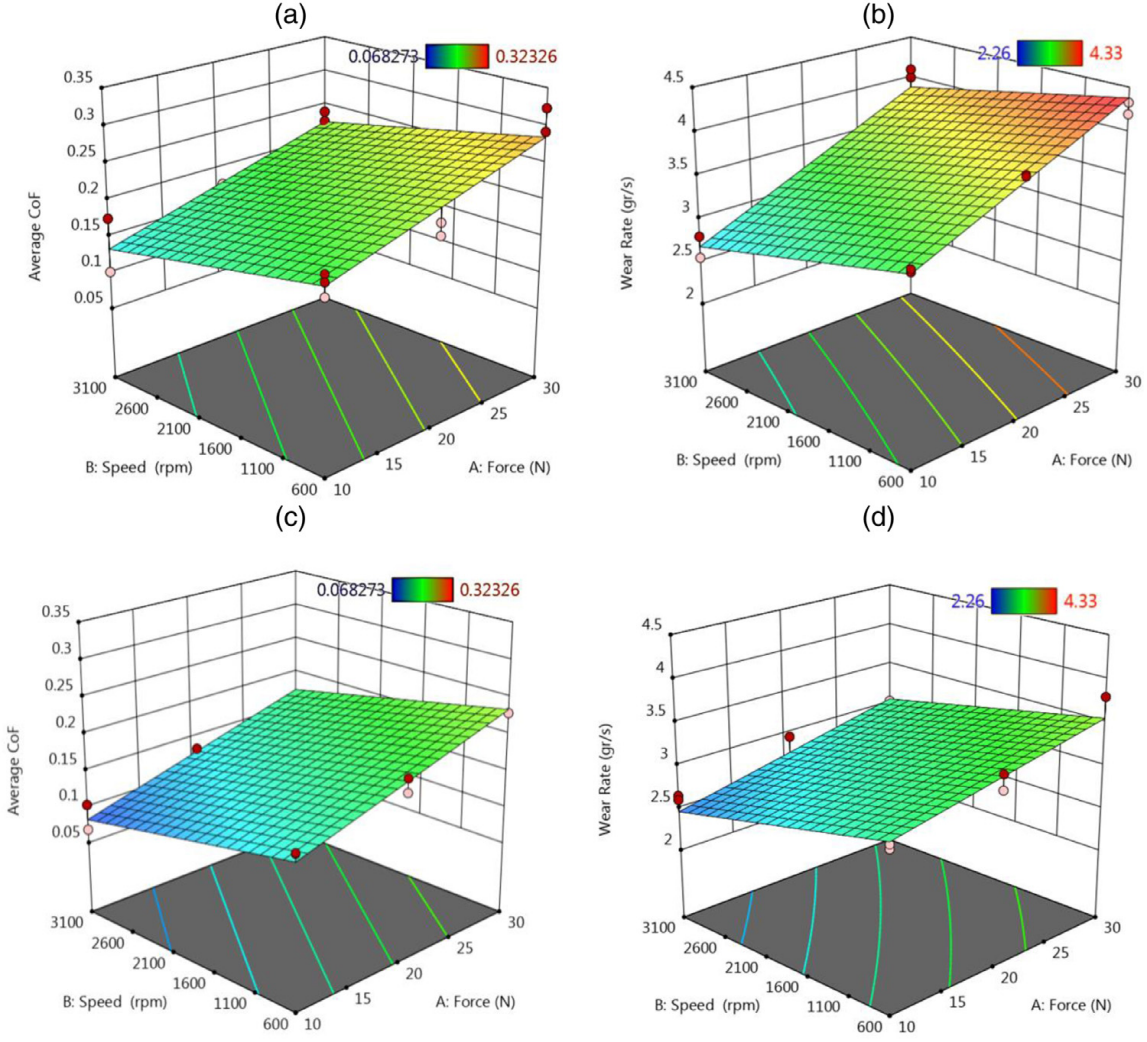
The surface plots of outputs (the average CoF and the total wear rate × 10^−4^) versus the speed and the force: (a) and (b) for the piston aluminum alloy and (c) and (d) for the nano-composite.

Finally, Fig. 8 illustrates the contour plots of outputs versus the speed and the force, for both material types, including the nano-composite and the piston aluminum alloy. Such results for surface plots could be seen in Fig. 9. Based on these figures, changes in the tribological behavior of the piston aluminum alloy were more severe than those of the nano-composite, since red regions could be observed in Fig. 8(a) and (b) and also Fig. 9(a) and (b).

 

## 2 Experimental Design, Materials and Methods

This investigation is on the wear behavior of piston aluminum alloys in the vehicle engine. The chemical composition of the studied material could be found in Table 5. Besides, the nano-composite specimen had 1% wt. of clay nano-particles, in addition to the mentioned composition in Table 5.

**Table 5:** The chemical composition (% wt.) of the aluminum alloy in the engine piston.

Al	Si	Cu	Mg	S	Ni	Fe	Zn	Mn
83.30	12.70	1.16	1.00	0.01	0.80	0.56	0.16	0.12

Initial cylindrical samples of aluminum alloys were casted in a permanent-mold casting process was performed. For the aluminum-matrix nano-composite, a stir-casting process was utilized for fabricating samples in addition to the preheating technique of nano-particles. More details of the sample fabrication are mentioned in the literature [12].

**Fig. 10:**
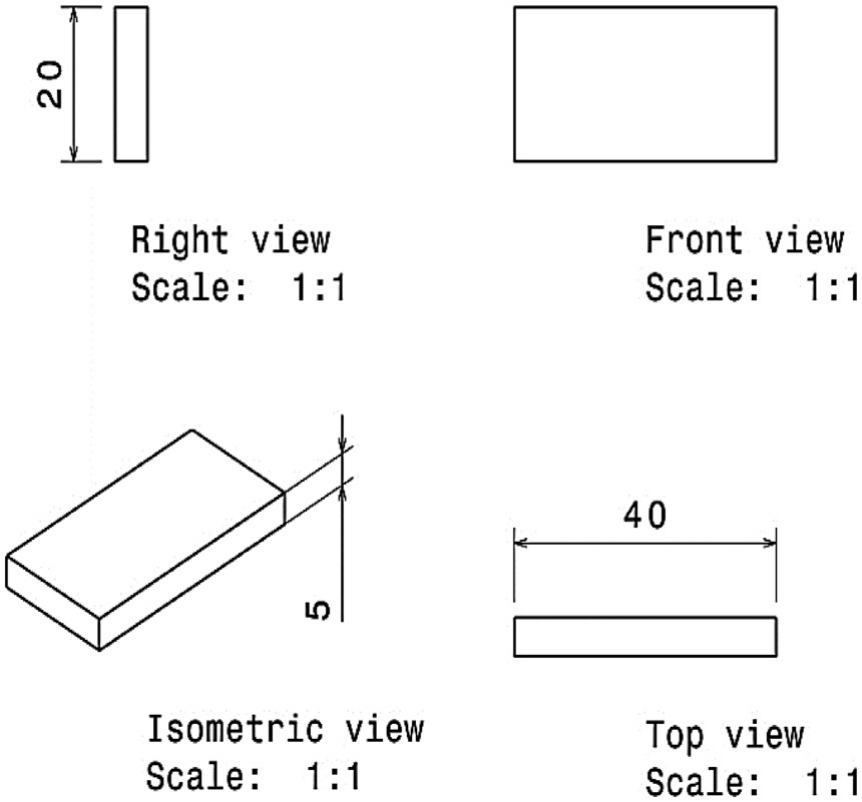
The dimension (in mm) of wear testing specimens.

**Fig. 11:**
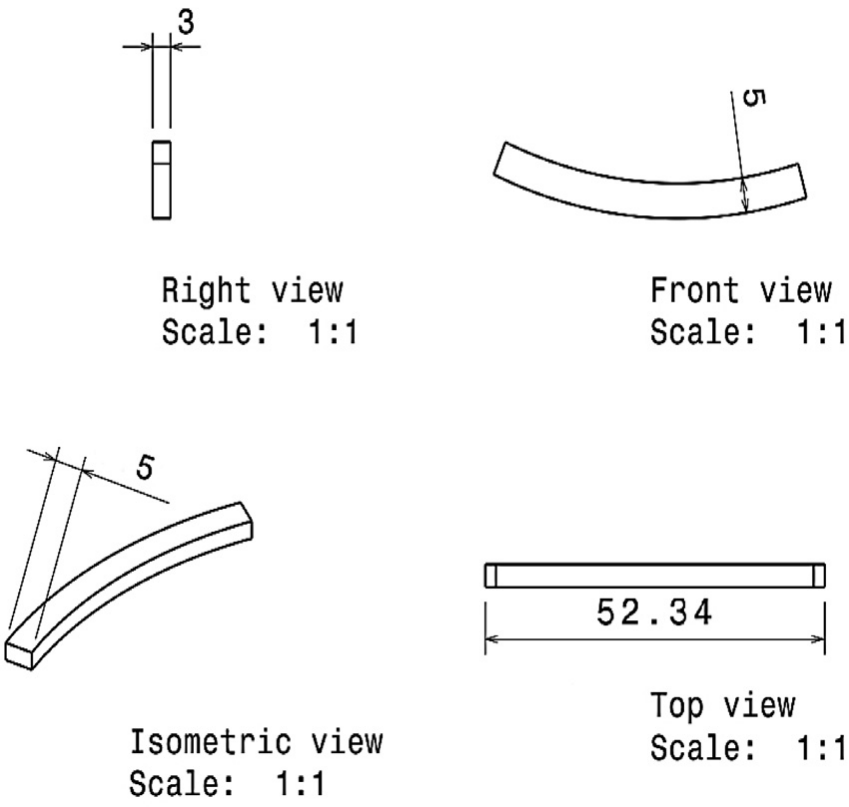
The dimension (in mm) of the ring abrasive system.

After casting, the sample was machined based on the mentioned geometry in Fig. 10. This specimen was under contact with the abrasive system, which was cut from a real piston ring. The piston ring material was a nodular cast iron (MF-116) with 35-45 HRC of hardness. The geometry of the abrasive system is depicted in Fig. 11.

The ASTM-G133 standard was considered for reciprocating wear testing. The testing distance was 500 m. The applied force was selected as 10, 20, and 30 N. The linear velocity was also 1 and 7 m/s, which was equal to 600 and 3600 rpm of the wear testing device. These values were based on the working conditions of the piston-ring component in the engine [6], [7], [8],^10^,^11^,^13^,14].

**Fig. 12:**
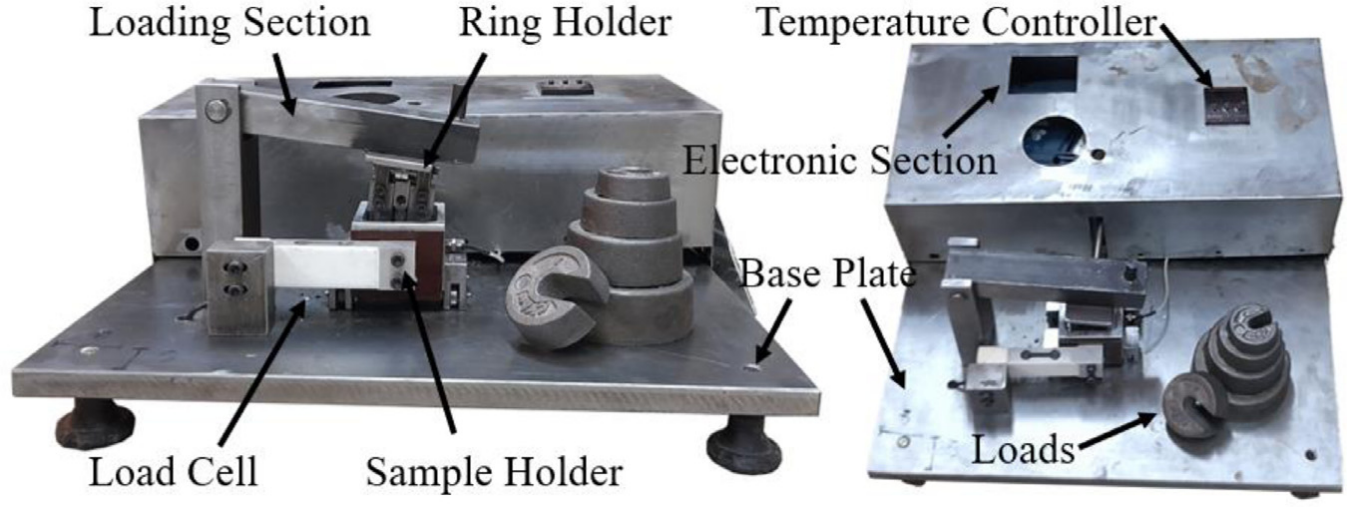
The device for reciprocating wear testing.

**Fig. 13:**
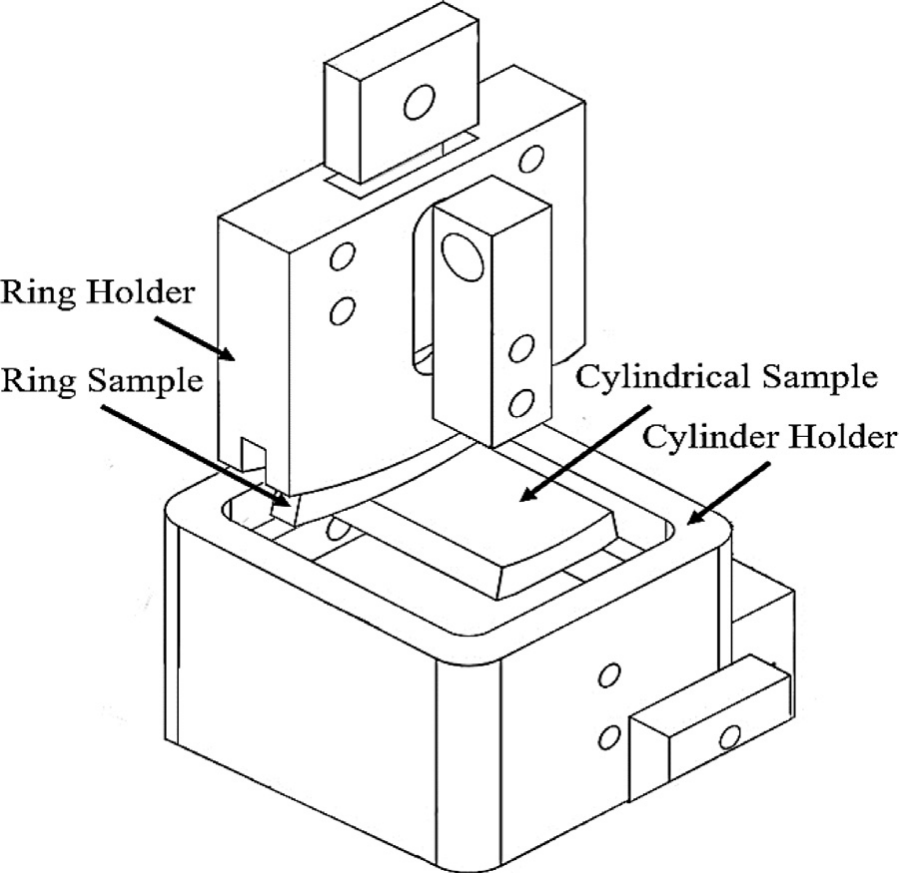
The wear mechanism in the testing device.

The testing device for the reciprocating wear behavior of the material could be seen in Figs. 12 and 13. Different parts of the testing device could be observed in these images, including the fixtures, the electronic unit, and the load cell for measuring the contact force and calculating the friction coefficient.

One temperature controller could be also found on the testing device. In this study, the temperature was considered as the room temperature of 25°C. The dry environment was also dry without oil or lubricant. In addition, the sample weight was measured during wear testing at the distance interval of 100 m, with the accuracy of 0.001 gr. For the total wear rate, the overall change in the weight was calculated over the whole duration of testing (through 500 m of the wear distance).

In order to analyze the experimental data on wear testing, a nonlinear regression technique by the Design-Expert software was utilized as the sensitivity analysis. For this objective, all parameters for inputs and outputs are demonstrated in Tables 6 and 7, respectively. It should be noted that two parameters of the force and the speed were numeric and the nano reinforcement was categorical. After the sensitivity analysis, the *P*-Value, the *F*-Value, and the coefficient of determination (*R*^2^) were reported. Notably, a higher *F*-value means higher effectiveness of the parameter. A *P*-Value higher than 0.05 (equal to 95% of the confidence level) means the parameter was effective. Lower than this value means that the variable effect was not significant. Finally, the *R*^2^ value demonstrates the fitness quality of the nonlinear equation of experimental data. More details could be found in the literature [15,16].

**Table 6:** The inputs for the sensitivity analysis of piston aluminum alloys.

Factor	Name	Units	Minimum	Maximum	Coded Low	Coded High	Mean	Std. Dev.
A	Force	N	10.00	30.00	-1 ↔ 10.00	+1 ↔ 30.00	18.70	8.15
B	Speed	rpm	600.00	3100.00	-1 ↔ 600.00	+1 ↔ 3100.00	1795.65	1276.88
C	Nano Reinforcement	-	0	1	0 ↔ without nano-particles	1 ↔ with nano-particles	Levels:	2.00

**Table 7:** The outputs for the sensitivity analysis of piston aluminum alloys.

Response	Name	Units	Observations	Minimum	Maximum	Mean	Std. Dev.	Ratio
R1	Average CoF*	-	23	0.0683	0.3233	0.1825	0.0590	4.73
R2	Total wear rate	gr/s	23	2.26 × 10^−4^	4.33 × 10^−4^	3.29 × 10^−4^	0.5733 × 10^−4^	1.92

⁎ CoF: Coefficient of friction.

As another issue, the coefficient of friction (CoF) was measured during wear testing. Then, an average value was calculated from all CoF values. Based on data in Table 7, the ratio of the maximum to minimum values for the average CoF was 4.73, which illustrated a wider scatter-band for obtained data, compared to that of 1.92 for the total wear rate.

## Ethics Statements

It is not generally applicable in the presented data.

## CRediT authorship contribution statement

**Mohammad Azadi:** Conceptualization, Methodology, Investigation, Validation, Writing – original draft, Writing – review & editing, Supervision. **Ali Shahsavand:** Methodology, Investigation, Validation, Data curation, Software, Visualization. **Mohammad Sadegh Aghareb Parast:** Methodology, Investigation, Validation, Data curation, Software, Visualization.

## Declaration of Competing Interest

The authors declare that there are no known competing financial interests.

## Data Availability

Reciprocating wear testing raw data on piston aluminum alloys (Original data) (Mendeley Data).
